# Impact of pharmacist insulin injection re-education on glycemic control among type II diabetic patients in primary health clinics

**DOI:** 10.1016/j.jsps.2021.04.028

**Published:** 2021-04-30

**Authors:** Selvakumari Selvadurai, Kit Yee Cheah, Min Wei Ching, Hanisah Kamaruddin, Xiao You Lee, Radhiatul Mardhiyah Ngajidin, Xian Hui Lee, Lina Mariana Mohd Ali

**Affiliations:** a Jabatan Kesihatan Wilayah Persekutuan Kuala Lumpur & Putrajaya, Jalan Cenderasari, Tasik Perdana, 50480 Kuala Lumpur, Wilayah Persekutuan Kuala Lumpur, Malaysia; b Centre for Clinical Trial, Institute for Clinical Research, National Institute of Health, Kompleks Institut Kesihatan Negara (NIH), No. 1, Jalan Setia Murni U13/52 Seksyen U13, Setia Alam, 40170 Shah Alam, Selangor, Malaysia

**Keywords:** Insulin injection technique, Re-education, Pharmacists, Counselling, Glycaemic control

## Abstract

**Background:**

Insulin injection technique re-education and diabetes knowledge empowerment has led to improved glycemic control.

**Objectives:**

To evaluate the impact of pharmacist’s monthly re-education on insulin injection technique (IT), lipohypertrophy, patients’ perception on insulin therapy and its effect on glycaemic control.

**Methods:**

This randomized controlled, multi-centered study was conducted among type 2 diabetics from 15 government health clinics. 160 diabetics with baseline HbA1_C_ ≥ 8% and unsatisfactory IT technique were randomized into control or intervention group. Control group received standard pharmacist counselling during initiation and at 4th month. Intervention group received monthly counselling and IT re-education for 4 months. Assessment of diabetes, IT knowledge, adherence and perception towards diabetes were conducted using validated study tools Insulin Treatment Appraisal Scale (ITAS) and Medication Compliance Questionnaire (MCQ)).

**Results:**

139 patients completed the study; control group (69), intervention group (70). In control group, all outcomes shown improvement except for patient’s perception. Mean HbA1_C_ decreased 0.79% ± 0.24 (p = 0.001). In intervention group, all outcomes improved significantly. HbA1c reduces significantly by 1.19% ± 0.10 (p < 0.001). Monthly re-education improved patient’s perception towards insulin therapy (ITAS score reduced 1.44 ± 2.36; p = 0.021). Between groups, interventional arm shown significantly better improvement in all outcomes. Improvement was shown in IT technique (+2.02 score; p < 0.001), medication adherence (+1.48 score; p < 0.001) and ITAS (−1.99 score; p = 0.037). Mean HbA1_C_ reduced an additional of 0.63% (p = 0.008) compared to control arm.

**Conclusion:**

Re-education is more effective in increasing adherence, reducing lipohypertrophy, improving injection technique and patient’s perception on insulin therapy, thereby providing better glycaemic control.

## 1 What is known and objective

According to the World Health Organization (WHO), the prevalence of diabetes has risen from 4.7% in 1980 to 8.5% in 2014.(Sarwar et al., 2010) The National Health & Morbidity Survey 2015 (NHMS) reported that the overall prevalence of diabetes mellitus (known and undiagnosed) among adults of 18 years and above in Malaysia was 17.5%, with 25.1% of known diabetic patients claiming to use insulin (Institute for Public Health (IPH), 2015). Correct insulin injection practices are important in diabetes management (Dolinar, 2009, Grassi et al., 2014). Insulin injection practices (standard of care) include proper storage of insulin, proper time of injection, correct injection technique (IT), rotation site, disposal of needle, management of hypoglycemia and side effects (Nakatani et al., 2013, Grassi et al., 2014). Correct IT is defined as one that reliably delivers medication into the subcutaneous space without leakage and minimal discomfort (Pledger et al., 2010). Correct IT is just as important as the type and dose of insulin delivered to achieve appropriate glycaemic control (Strauss et al., 2002b). Good technique includes correct site rotation as well as not injecting into lipohypertrophy areas but the ITQ survey using UK data showed that 75% of patients did not practice site rotation and 54% of patients reported having lipohypertrophy at some point in their life, with 28% injecting into liohypertrophy sites (Coninck et al., 2010). Many patients either do not remember having been exposed to the information or the information was not conveyed to them at all (Coninck et al., 2010).

Despite considerable advances in technologies and therapies over the past decade, the way people inject has not improved (Strauss et al., 2002a, Coninck, et al., 2010). Re-education in the insulin injection technique has led to an improvement in glycemic control in insulin-treated diabetic patients, especially in those with poor understanding of the insulin injection technique, as the decrease in HbA1c of these patients was significantly large, at 7.62 ± 0.20% to 6.71 ± 0.21%. (Nakatani et al., 2013). Targeted individualized training in IT is associated with improved glucose control, greater satisfaction with therapy, better and simpler injection practices and possibly lower consumption of insulin (Grassi et al., 2014).

Lipohypertrophy (LH) is an abnormal accumulation of fat underneath the surface of the skin and is most commonly seen in people who receive frequent and multiple daily injections (Ann Pietrangelo and Ana Gotter, 2017). Repeated insulin injections in the same location can cause fat and scar tissue to accumulate (Ann Pietrangelo and Ana Gotter, 2017). Factors causing LH which include needle change frequency, change of site frequency, and duration of insulin use (Dolinar, 2009), are usually taught during insulin injection technique counseling. Injection into these areas may result in variability in absorption, bruising, bleeding and erratic glycemic control (Pledger et al., 2010). Poor insulin injection technique increases chances of developing LH, which consequently would lead to erratic glycemic control (Vardar and Kizilci, 2007).

It is crucial to do the visual and physical examinations to look for deformities at the injection site (Strauss et al., 2002b). Individuals should be taught to examine their own injection sites and how to detect lipohypertrophy (FIT UK Forum for Injection Technique, 2016). Diabetes related knowledge showed significant improvement after education program thus improve the quality of life in diabetic patients (Milenkoviæ and Gavriloviæ, 2004).

Pharmacists’ role has expanded over the years. It is important for pharmacist to identify patients’ needs and to work together with other healthcare team members to improve patients heath status, clinical outcomes and quality of life (Lindenmeyer et al., 2006). Thus so far, it is known that insulin technique re-education can improve glycaemic control, however no study in Malaysia has investigated on the effect of monthly insulin injection re-education on the insulin technique of patients with poor glucose control compared with those who had only standard counselling. No controlled studies have been done to look at other outcomes such as lipohypertrophy and patients’ perception of insulin therapy. Therefore, the aim of this study was to evaluate the impact of pharmacist’s monthly insulin injection re-education on injection site rotation and its effect on lipohypertropy, patients’ perception of insulin therapy, and glycaemic control.

## 2 Methods

### 2.1 Study design and setting

This randomized controlled, multi-centered study was conducted within six months follow-up period, among type 2 diabetic patients from 15 government health clinics from Jabatan Kesihatan Wilayah Persekutuan Kuala Lumpur & Putrajaya, Malaysia. The recruitment and data collection were performed between April 2017 to September 2017. The study was approved by the Medical Research and Ethics Committee, Ministry of Health Malaysia (Ref No: NMRR-16-1591-28767).

### 2.2 Patient recruitment

Type 2 diabetic patients who are Malaysian citizens aged 18 and above with baseline HbA1_C_ level ≥ 8% and having unsatisfactory insulin injection technique after self-injecting insulin using insulin pens for at least one year were recruited. A 17-point structured insulin technique (IT) checklist adapted from the Malaysia’s Ministry of Health insulin counselling checklist were used to screen the patients with unsatisfactory insulin injection technique (Pharmaceutical Services Division, 2014). Seven core counselling points from this checklist were pre-identified by the study investigators comprising of pharmacists to comprise the main aspects of correct insulin injection technique. Patients who were unable to perform these seven points correctly were deemed to have poor insulin injection technique. Patients with end stage renal failure (ESRF), gestational diabetes mellitus (DM), on chronic steroids and who has participated in any other pharmacy diabetes educational programs were excluded from the study.

**Fig. 1:**
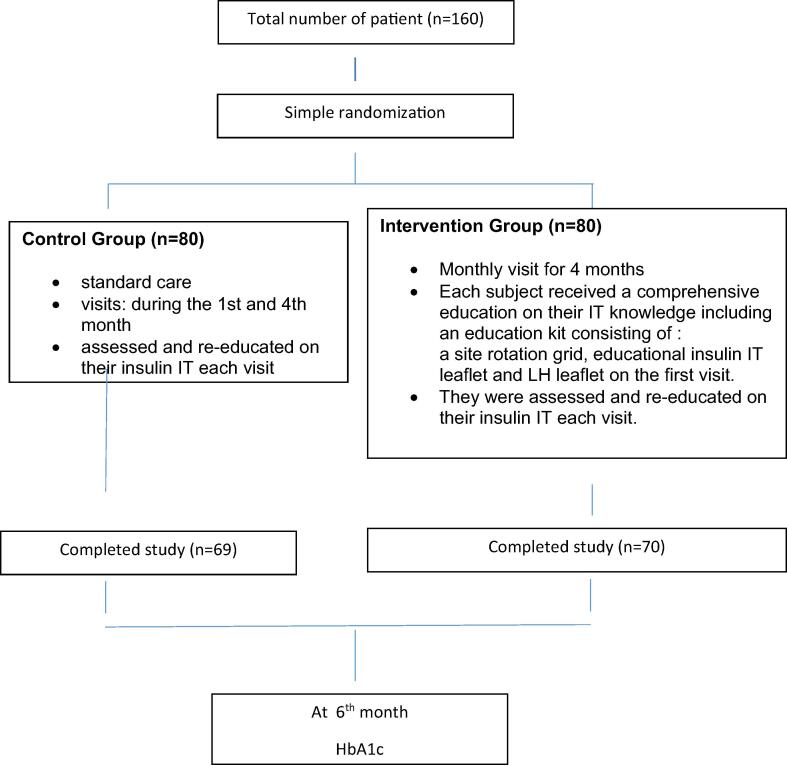
Trial flow diagram.

Eligible patients who consented to participate in the study was categorized to control group (CG) or intervention group (IG) randomly. Simple randomization allocation sequence was generated using a computer programme and provided to each study centre prior to commencement of the study. Allotment of number of recruitments per health clinics was calculated based on workload and number of patients serviced. This was an open-label study in which patients and study investigators who are also technique assessors were not blinded from the study treatment. All patients were individually interviewed to obtain socio-demographic information, medication history and clinical history of diabetes. Pharmacist-assisted assessments of diabetes, medication knowledge, adherence to medication, insulin injection technique and perception about diabetes were conducted during recruitment using study tool. Please refer to Fig. 1 for a flow diagram of trial recruitment.

### 2.3 Study tool

The IT technique score was based on the 17-point structured insulin technique (IT) checklist adapted from the Malaysia’s Ministry of Health insulin counselling checklist, where each point correctly done by the patient gives one score, resulting in a maximum total score of 17 (Pharmaceutical Services Division, 2014) The questionnaire was validated with a pilot study using 20 patients. Patient’s perception on diabetes and its treatment was assessed using validated Insulin treatment appraisal scale (ITAS) (Snoek, Skovlund and Pouwer, 2007). The appraisal consists of 20 items, with 16 negatively worded statements and 4 positively worded statements. Subjects were required to indicate on a 5-point Likert scale on how likely they agree with each statement, with choices ranging from “strongly agree” to “strongly disagree”. The lower the ITAS score, the more positive perception towards insulin treatment. Patients medication adherence level were assessed using Medication Compliance Questionnaire (MCQ) (Sufiza Ahmad et al., 2013). Higher MCQ score denotes better adherence. Lipohypertrophy was identified from injection site via palpation by trained pharmacist. The study tools were administered by trained data collectors comprising of pharmacists. Face-to-face interviews with study patients were conducted in Malay language.

### 2.4 Control group (CG)

Patients in the CG received standard pharmacist counselling during the study period. They were counselled on insulin injection technique including lipohypertrophy (LH) physical examination during recruitment and at the end of the study. At the 4th month, MCQ, ITAS, ITQ and lipohypertrophy physical examination were assessed.

### 2.5 Intervention group (IG)

Patients in IG were given intensive re-education during their monthly medication acquisition visits for 4 months. Each patient was given comprehensive education on their injection technique knowledge technique including LH physical examination and received an education kit consisting of a site rotation grid, educational insulin injection technique and LH leaflet during recruitment. They were assessed and re-educated on their insulin injection technique and medication adherence on each visit. At the 4th visit, MCQ, ITAS, ITQ and lipohypertrophy physical examination were assessed.

### 2.6 Outcomes measures

The primary outcome measures were changes in adherence, injection technique, ITAS score and improvement of lipohypertrophy. The secondary outcome measure was improvement in HbA1c after 6 months as HbA1c was sampled every 6 months in most primary healthcare setting. Table 1 shows the summary of outcome measure assessment and intervention performed at each visits.

**Table 1:** Summary of outcome measure assessment and intervention.

**Outcome**	**Tool**	**Control Group** Measurement timeline (month)					**Intervention Group** Measurement timeline (month)				
		1st	2nd	3rd	4th	6th	1st	2nd	3rd	4th	6th
HbA1c		**√**				**√**	**√**				**√**
Medication adherence	Medication Compliance Questionnaire (MCQ) (23)	**√**			**√**		**√**	**√**	**√**	**√**	
Perception on diabetes and treatment	Insulin Treatment Appraisal Scale (ITAS) (22)	**√**			**√**		**√**			**√**	
Injection technique assessment	Insulin technique checklist, KKM	**√**			**√**		**√**	**√**	**√**	**√**	
Lipohypertrophy physical examination		**√**			**√**		**√**	**√**	**√**	**√**	
Education kit							**√**				

### 2.7 Statistical analysis

Collected data was analysed using The Statistical Package for the Social Sciences (SPSS, Version 22 Inc., Chicago, IL, USA). Comparisons were made between baseline data and final assessments using the appropriate statistical tools with per-protocol analysis. Baseline demographic for all enrolled patients were reported, however only patients who have completed the study and not lost to follow-up were analysed for outcomes analysis. For normally distributed continuous data, paired *t*-test was used for within group analysis whereas Student’s *t*-test was used for between group analysis. Chi-square test was used for nominal variables. The normality and assumptions for statistical tests were performed before applying the statistical tests. The p-value is significant at p < 0.05.

## 3 Results and discussion

Out of the total 160 recruited patients, 80 patients were randomized to the control group and another 80 into the intervention group. A total of 139 patients completed the study, control group (69), intervention group (70). Twenty-one (13%) patients did not complete the 4 visits and were lost to follow-up. Fig. 1 shows the flow diagram of the study. The baseline demographics were presented in Table 2. There were no significant difference in the demographics and clinical presentations of the patients recruited in this study.

**Table 2:** Baseline demographic and clinical characteristics of participants (n = 160).

Characteristics	Control Group (n = 80)	Intervention Group (n = 80)	p-Value
Age, mean ± SD*	58.16 ± 9.06	56.46 ± 9.44	0.247

Education			0.872
Primary	19 (23.8%)	23 (28.8%)	
Secondary	34 (42.5%)	34 (42.5%)	
Tertiary	19 (23.8%)	16 (20.0%)	
No formal education	8 (8.0%)	7 (7.0%)	

Monthly Income			0.863
< RM 2000	39 (48.8%)	36 (45.0%)	
RM 2000–5000	31 (38.8%)	36 (45.0%)	
RM 5000–10,000	9 (11.2%)	7 (8.8%)	
RM 10,000	1 (1.2%)	1 (1.2%)	
Insulin duration, mean ± SD* (year)	4.84 ± 5.59	4.12 ± 3.49	0.330

Total daily insulin injections			0.784
One	17 (21.2%)	21 (26.6%)	
Two	30 (37.5%)	27 (34.2%)	
Three	5 (6.2%)	3 (3.8%)	
Four	28 (35.0%)	28 (35.4%)	
HbA1c, mean ± SD* (%)	10.35 ± 1.70	10.31 ± 1.47	0.858
Detection of lipohypertrophy	17 (21.3%)	24 (30.0%)	0.205
Adherence level, mean ± SD* (score)	25 (1.93)	25 (2.05)	0.133
Perception (ITAS)	51.40 (10.21)	51.29 (8.86)	0.943

* SD = standard deviation

### 3.1 Outcome measures within group

The changes in the outcomes measures within control and interventional groups were presented in Table 3. In the control group, all primary and secondary outcomes showed significant improvement after the study except for patient perception towards insulin treatment and incidence of lipohypertrophy. Mean HbA1c decreased 0.79% ± 0.24 after standard pharmacist counselling. However, there is a decrease in ITAS score (−0.21 ± 0.14) at the end of the study although it is not significant. There is a non-significant reduction of 7.4% (p > 0.05) in the presence of lipohypertrophy in the control group.

**Table 3:** Outcome changes from baseline within control and intervention groups.

**Variable**	**Assessment**	**Control Group (n = 69)**	**Intervention Group (n = 70)**
Insulin injection technique	Pre	12.75 ± 2.70	12.24 ± 1.46
(score, mean ± SD)	Post	15.11 ± 3.65	16.53 ± 0.94
p value (within group)		<0.001#	<0.001#

Medication adherence	Pre	25.14 ± 1.93	24.66 ± 2.05
(score, mean ± SD)	Post	26.04 ± 1.40	26.97 ± 0.94
p value (within group)		<0.001#	<0.001#

Patient's perceptions (ITAS)	Pre	51.40 ± 10.12	51.29 ± 8.86
(score, mean ± SD)	Post	51.19 ± 9.98	49.85 ± 11.22
p value (within group)		0.806#	0.021#

Presence of lipohypertrophy (LH)	Pre	17 (21.3)	24 (30.0)
n (%)	Post	10 (13.9)	14 (18.9)
p value (within group)		0.07*	0.02*

HbA1c	Pre	10.35 ± 1.70	10.32 ± 1.46
(%, mean ± SD)	Post	9.56 ± 1.94	9.13 ± 1.56
p value (within group)		0.001#	<0.001#

# Paired-*t* test * Chi-square

All primary and secondary outcomes improved significantly in the intervention group. HbA1c reduced significantly by 1.19% ± 0.10 (p < 0.001) after intervention. Intensive counselling was able to improve patient’s perception towards insulin therapy with reduction of ITAS score by 1.44 ± 2.36. Number of patients with lipohypertrophy also reduced significantly by 11.9% (P = 0.02).

### 3.2 Outcome measures between group

Compared to the control group, patients in the intervention group had shown significantly better improvement in outcomes. HbA1c reduced by an additional 0.39% to 1.13% compared to 0.74% in the control group. Insulin injection technique and adherence also reported significantly better outcome. ITAS score had improved significantly in the intervention group compared to control group who had a slightly more negative perception towards insulin therapy, with significant difference score by −1.99 (mean ITAS score changes of −1.86 in the intervention group compared to + 0.13 in the control group). However, there was no statistically significant difference in incidence of lipohypertrophy between control and intervention group although clinically more patients had reported improvement in the intervention group.

In Malaysia, patients are usually given insulin injection counselling by pharmacists after insulin initiation by the physician. Due to the lack of human resources and time constraint, it is not compulsory for patients to undergo repeated insulin counselling unless requested by their physician if the patient’ blood sugar shows no sign of improvement despite treatment optimization. Hence, this study was designed to observe the effects if any, on patients with uncontrolled blood sugar, after they undergo additional insulin counselling delivered by pharmacists in a primary care setting. Most studies were done comparing between pharmacist-led interventions and usual care involving medical and nursing staff (Stading et al., 2009, Butt et al., 2016).

As observed from Table 4, both control and intervention groups showed statistically significant improvement in the measured outcomes of their HbA1c, insulin injection technique, medication adherence and patients’ insulin perception scores as well as presence of lipohypertrophy. However, a greater improvement in blood sugar control can be seen from patients in the intervention group as they displayed a greater reduction in HbA1c than those in the control group. This finding is better than that of a similar study with a similar length of follow-up done by Cani et al (Cani et al., 2015), where those in the group receiving monthly counselling by the clinical pharmacist had a mean HbA1c reduction of 0.6%. However, the fact that the patient population of that study was from a teaching hospital and may have more underlying co-morbidities affecting their glycaemic control may be the cause of this occurrence. In any case, the reduction of HbA1c in this study is similar to that of a previous one done on the same population, participating in a pharmacist-led diabetic intervention programme where the mean reduction of HbA1c is 1.0% (You et al., 2015). Nonetheless, the results in that study were of a smaller sample and had no control group. Standard pharmaceutical care which is the control group in this study, showed reduction in HbA1c, albeit a significantly smaller reduction compared to the intervention group. This study showed that additional pharmacist-led intervention further enhanced the standard pharmaceutical care in helping diabetic patients in primary care to achieve optimal glycaemic control in the current local population. The meaningful reduction in HbA1c of 1.0% on these patients in a primary care setting showed that such an intervention on glycaemic control might give a much more reduction in HbA1c if this study is replicated in diabetic patients in a hospital setting.

**Table 4:** Outcome changes from baseline between control and intervention groups.

**Variable (changes from baseline)**	**Control Group (n = 69)**	**Intervention Group (n = 70)**	**p value**
Insulin injection technique (score, mean ± SD)	2.25 ± 2.50	4.27 ± 2.63	<0.001#
Medication adherence (score, mean ± SD)	0.84 ± 1.23	2.32 ± 1.93	<0.001#
Patient's perceptions (ITAS) (score, mean ± SD)	0.13 ± 4.30	−1.86 ± 6.75	0.037#
Improvement of lipohypertrophy (LH) n (%)	7 (41.2)	10 (41.7)	0.792*
HbA1c (%, mean ± SD)	−0.74 ± 1.54	−1.13 ± 1.25	0.008#

# Independent *t* test * Chi-square

The increase in insulin technique injection scores in both groups of patients pre-intervention and post-intervention proves that patients’ knowledge regarding correct insulin technique injection improved after pharmacist counselling. This result is similar to a recent cohort study done on Polish diabetic patients where professional education resulted in better insulin injection technique as well as glycaemic control. (Gorska-Ciebiada, Masierek and Ciebiada, 2020) One study on insulin injection technique education outcome in Malaysian patients was published, where there was a 0.82% reduction in participants’ HbA1c 3 months after they received correct insulin injection technique education (Ahmad et al., 2016). As this study was only followed-up for 3 months post-intervention, it can be postulated that a greater reduction in HbA1c similar to the current study may be seen if the participants were followed up for a longer duration, which is proven by the current study with a 6-month follow-up period. This could suggest that it is necessary to continuously assess insulin users’ injection technique on a regular basis for all the insulin users by the pharmacists.

A recent literature review looking on insulin adherence and glycaemic control association have shown that most studies on insulin adherence is associated with better glycaemic control (Doggrell and Warot, 2014). However, another review by Doggrell et al concluded that there is little or no evidence that an additional intervention by a doctor or pharmacist discussing insulin adherence does improve the measured adherence (Doggrell and Chan, 2015). As one of the most common barriers to insulin non-adherence which indirectly involve insulin injection technique is fear of hypoglycaemia, injection-site reactions and lack of adequate injection instructions,(Peyrot et al., 2012, Petznick, 2013, Sarbacker and Urteaga, 2016) this study was designed such that no adherence counselling was given but adherence at baseline and post-intervention was measured to see if improved knowledge on insulin injection technique could affect adherence. Results from this study proved that there was an improvement in medication adherence after both standard pharmaceutical care and greater adherence after comprehensive education regarding insulin injection technique was given, it can be postulated that better injection technique leading to less hypoglycaemia frequency and less injection-site reactions may indirectly increase patients’ adherence towards insulin therapy.

Apart from that, the decrease in ITAS scores in the intervention group showed that patients had more positive appraisal towards insulin therapy after comprehensive insulin injection education. Several studies have shown that insulin perception is modifiable as insulin naïve patients often have a higher negative insulin appraisal compared to insulin users (Chen et al., 2011, Gulam et al., 2017, Holmes-Truscott et al., 2018, Hermanns et al., 2010). One question on the ITAS scale which was scored significantly better after intervention was related to painful injection; hence it could be hypothesized that improved injection technique decreased the frequency of painful injections. It is not known why there was a minimal increase in negative insulin appraisal in the control group, but perhaps a greater increase in injection technique scores was needed to give a positive effect towards insulin appraisal. This result is interestingly reflected in Malgorzata’s study as well where a decrease in the sensation of pain was observed in patients after receiving professional education. (Gorska-Ciebiada, Masierek and Ciebiada, 2020)

Lastly, the decrease in percentage of patients having LH post-intervention showed that presence of LH may have decreased due to better insulin injection technique. Identified risk factors for LH included needle reuse and failure to rotate injection site.(Gentile et al., 2020) Hence, the decrease in this study could be due to better understanding of the insulin injection technique after re-education. To date, there is no study looking into presence of LH post-intervention by any healthcare professional in Malaysia but as mentioned above, Grassi et al showed a significant mean reduction in HbA1c of 0.58% after 3 months post insulin injection technique counseling (Grassi et al., 2014). The better reduction of 1.0% in HbA1c in this study could be due to a longer follow-up of six months instead. However, the non-significant improvement in presence of LH between the control and intervention group could be due to insufficient time of follow-up.

A recent meta-analysis on the impact of pharmacist based diabetes educational interventions has found that all interventions significantly reduced HbA1c, but there was no statistical evidence to prove that one was better than the other.(Bukhsh et al., 2018) However, the same paper concluded that pharmacist based diabetes education plus pharmaceutical care showed the highest impact on HbA1c reduction and other clinical outcomes (Bukhsh et al., 2018). The significant improvements in the various aspects of insulin injection technique, medication adherence and overall insulin perception of the intervention group in this study can thus be hypothesized to be attributed to the increased frequency of counselling given by the pharmacists with an additional comprehensive education on their injection technique knowledge technique, leading to a reduction in the patients HbA1c. Furthermore, in this study, the patients in the intervention group had a significantly greater reduction in HbA1c as compared to those in the control group, thus displaying statistical evidence that pharmacist led diabetes education and pharmaceutical care had a much higher impact on HbA1c reduction and further enhancing the standard pharmaceutical care practices.

## 4 Limitations

Our study had shown favourable impact from insulin injection re-education. However, the findings may not be fully representative of the general diabetes population since it was conducted in the primary healthcare setting and excluding patients who were in the Diabetes Medication Therapy Adherence Clinic (DMTAC) programme conducted by pharmacists. The short duration of the study also was a limitation to measure the sustainability of the favourable outcomes.

## 5 What is new and conclusions

As such from the findings of this study, a regular or a compulsory re-assessment of insulin injection technique session is to be implemented. All newly started patients on insulin must undergo a second IT assessment by the pharmacists. Pharmacists are recommended to check on patient’s LH which should be a part of their insulin injection technique counselling module. However, presence of LH should be checked physically by the pharmacist during their counselling sessions with the diabetic patients. Hence, pharmacists should be taught or trained to detect LH physically as part of their counselling module.

There are many other factors which can affect diabetic control apart from insulin injection technique such as diet and physical activity, all of which were not controlled in this study. Perhaps controlling for such factors in addition to improving insulin injection technique could lead to a better improvement of glucose control of more than 1.0% HbA1c.

Insulin injection technique re-education helps to facilitate improvement in glycaemic control by improving diabetes patient’s medication adherence, insulin injection technique and injection site inspection as well as perception towards insulin therapy. Monthly re-education proves to be a more effective measure to help diabetes patient’s to achieve their glycaemic goal.

## Declaration of Competing Interest

The authors declare that they have no known competing financial interests or personal relationships that could have appeared to influence the work reported in this paper.
